# Whole-genome sequencing and secondary metabolite exploration of the novel *Bacillus velezensis* BN with broad-spectrum antagonistic activity against fungal plant pathogens

**DOI:** 10.3389/fmicb.2024.1498653

**Published:** 2025-01-03

**Authors:** Yanli Zheng, Tongshu Liu, Ziyu Wang, Xu Wang, Haiyan Wang, Ying Li, Wangshan Zheng, Shiyu Wei, Yan Leng, Jiajia Li, Yan Yang, Yang Liu, Zhaoyu Li, Qiang Wang, Yongqiang Tian

**Affiliations:** ^1^School of Biological and Pharmaceutical Engineering, Lanzhou Jiaotong University, Lanzhou, Gansu, China; ^2^State Key Laboratory of Crop Stress Adaptation and Improvement, School of Life Sciences, Henan University, Kaifeng, China; ^3^Academy for Advanced Interdisciplinary Studies, Henan University, Kaifeng, China

**Keywords:** green pesticides, food safety, Plant Growth-Promoting Rhizobacteria (PGPR), antimicrobial agents, non-ribosomal peptides

## Abstract

The utilization of chemical pesticides recovers 30%−40% of food losses. However, their application has also triggered a series of problems, including food safety, environmental pollution, pesticide resistance, and incidents of poisoning. Consequently, green pesticides are increasingly seen as viable alternatives to their chemical counterparts. Among these, Plant Growth-Promoting Rhizobacteria (PGPR), which are found within plant rhizosphere, stand out for their capacity to stimulate plant growth. Recently, we isolated a strain, BN, with broad-spectrum antimicrobial activity from the rhizosphere of *Lilium brownii*. Identification revealed that this strain belongs to the species *Bacillus velezensis* and exhibits significant inhibitory effects against various fungal plant pathogens. The complete genome sequence of *B. velezensis* BN consists of a circular chromosome with a length of 3,929,791 bp, includes 3,747 protein-coding genes, 81 small RNAs, 27 rRNAs, and 86 tRNAs. Genomic analysis revealed that 29% of the genes are directly involved in plant growth, while 70% of the genes are indirectly involved. In addition, 12 putative biosynthetic gene clusters were identified, responsible for the synthesis of secondary metabolites, such as non-ribosomal peptides, lanthipeptides, polyketides, siderophores, and terpenes. These findings provide a scientific basis for the development of efficient antimicrobial agents and the construction of biopesticide production platforms in chassis cells.

## 1 Introduction

The increasing frequency and severity of plant diseases present growing threats to global food security, biodiversity and agricultural production. These diseases reduced crop yields and caused ecological damage, with pathogens and pests alone accounting for up to 41% of global crop losses, costing the global economy over $220 billion annually (Savary et al., 2019; Singh B. K. et al., 2023). Crops are vulnerable to infections from a diverse array of pathogens, encompassing bacteria, fungi, oomycetes, viruses, and nematodes. Historically, pesticides have been the predominant method for managing plant diseases. Nevertheless, the rising resistance of these pathogens, coupled with the substantial environmental pollution caused by chemical usage, poses a grave risk to public health (Tang et al., 2021). In the rhizosphere of terrestrial plants, a diverse array of indigenous beneficial bacteria engage in close interactions with plant roots. These bacteria contribute to plant growth and bolster stress resistance through diverse mechanisms (Liu et al., 2020), presenting a promising alternative to traditional pesticides.

Indigenous bacteria consortia have a wide range of applications across multiple fields. Through isolation and optimization, they can eliminate heavy metals from wastewater, enhance soil ecological functions, and promote plant growth (Gu et al., 2024; La et al., 2024; Yan et al., 2024). However, the complex interactions among these microorganisms, along with strong environmental dependencies, make artificial regulation challenging. As a result, synthetic microbial communities (SynCom) have gained considerable attention in plant protection (Qiao et al., 2024; Wang et al., 2024).

SynCom are artificially created by combining two or more bacteria with distinct classifications, genetic traits, and functional characteristics. Research has shown that a SynCom, SSC8, composed of eight *Pseudomonas* can enhance the growth and disease resistance of watermelon (Qiao et al., 2024). A SynCom-mix7 was developed using seven *Pseudomonas*, significantly improving the resistance of *S. babylonica* to *P. versicolora* (Wang et al., 2024). These indicates that SynCom primarily consist of infraspecies, as interspecies combinations may lead to competition among microbes, making stable coexistence difficult.

To address this issue, researchers have proposed strategies and methods for artificial spatial isolation, successfully achieving interspecies microbial communities involving *E. coli, S. cerevisiae*, and *C. glutamicum* (Wang L. et al., 2022). Additionally, genome analysis of individual Plant Growth-Promoting Rhizobacteria (PGPR) can be employed to construct engineered strains with clear pathways that are artificially controllable, which can then be mixed with other strains. By designing the engineered strains, it becomes possible to create adjustable and stable dual- or multi-strain combinations (Fedorec et al., 2021).

*Bacillus* species, known as PGRP, are renowned for their abundant secretion of biologically active molecules, predominantly secondary metabolites with molecular weights under 2.5 KDa, which possess the ability to suppress the growth of plant pathogens (Jayakumar et al., 2011; Ruparelia et al., 2022). Although these molecules are not essential for bacterial growth or replication, they provide a substantial selective advantage upon the producing strain. This repertoire of bioactive molecules includes lipopeptides, antibiotics, enzymatic degraders, iron chelators, polyketides, non-ribosomal peptides, ribosomal peptides, volatile organic compounds (VOC), and specific plant hormones, all of which demonstrate antifungal, antibacterial, and antiviral properties, concurrently promoting plant growth (Grady et al., 2016; Ruparelia et al., 2022). Furthermore, the spore-forming ability of *Bacillus* species renders ideal candidate for developing highly efficacious biopesticide products (Ongena and Jacques, 2008; Zhao et al., 2023). To date, a number of *Bacillus* strains have been successfully commercialized as biocontrol agents, such as *Bacillus velezensis* FZB42 (ABiTEP, GmbH, Berlin, Germany), and *Bacillus pumilus* (QST2808; AgraQuest, Inc., Davis, California, USA) (Hèlène et al., 2011; Pérez-García et al., 2011).

Between 1999 and 2000, researchers successfully isolated two strains, CR-14b and CR-502^T^, from soil samples collected at the mouth of the Vélez River at Torre del mar in the province of Málaga, southern Spain (Ruiz-García et al., 2005). Phenotypic and phylogenetic analyses suggested a close relationship of these strains to *Bacillus subtilis* and *Bacillus amyloliquefaciens*. However, digital DNA-DNA hybridization (dDDH) revealed <20% sequence similarity with any known species within the *Bacillus* genus, which warranted their classification as a novel species, *Bacillus velezensis* (*B. velezensis*) (Dunlap et al., 2016; Ruiz-García et al., 2005; Wang et al., 2008). *B. velezensis*, an aerobic, Gram-positive bacterium, is capable of forming endospores and producing a wide range of secondary metabolites with broad-spectrum antibacterial activity, as well as its capacity to promote plant growth (Rabbee et al., 2019). Consequently, *B. velezensis* has seen widespread application in the biological control of plant diseases. At present, various strains of *B. velezensis* are being employed in the management of plant diseases. For instance, *B. velezensis* JB0319 helps lettuce to overcome salt stress and promote growth by enriching nitrogen-fixing bacteria (Bai et al., 2023). Another strain, *B. velezensis* BAC03, enhances the growth of several crops, including beets, carrots and cucumbers (Meng et al., 2016).

Among plant-associated *Bacillus* species, *B. velezensis* stands out as a paradigmatic organism due to its significant role in promoting plant growth and suppressing plant pathogens. Its primary mechanisms include producing antimicrobial substances to antagonize pathogens, competing for ecological niches and nutrients, and activating induced systemic resistance (ISR) of plants, offering a multifaceted approach to enhance plant growth and yield (Ongena and Jacques, 2008). Notably, *B. velezensis* allocates over 10% genome content to the synthesis of specialized secondary metabolites. These secondary metabolites have a unique ability to detect signaling molecules released by soil competitors, such as the siderophore pyochelin produced by *Pseudomonas aeruginosa*, which allows it to modulate its metabolic activities and deploy a range of defensive strategies (Andrić et al., 2023). Furthermore, the metabolites can significantly enrich plant-beneficial indigenous bacteria, for instance, *Pseudomonas stutzeri* in the cucumber rhizosphere, promoting the formation of stable mixed biofilms in the crop rhizosphere and conferring beneficial effects to the crops (Sun et al., 2022).

In this study, we introduce the complete genome sequence of the *B. velezensis* BN for the first time and demonstrate its broad resistance to various fungal plant pathogens. Our work not only elucidated the structural features of the *B. velezensis* BN genome but also delves into its functional genes, particularly those associated with promoting plant growth, utilizing rigorous whole-genome sequencing and analytical approaches. Furthermore, we have identified a suite of pivotal antimicrobial secondary metabolites. These findings have laid the foundation for the developing efficient microbial agents and constructing high-yield cell factories for value-added products.

## 2 Materials and methods

### 2.1 Isolation and culture of BN strain

The bulbs of *Lilium brownii* (lily) were collected from Lintao County, Gansu Province. First, the bulbs were rinsed with sterile water for 1 min, repeating the process two to three times, and allowed to air dry. Second, the bulbs underwent disinfection with 75% ethanol and 3% sodium hypochlorite, followed by three rinses with sterile water to eliminate any residual ethanol and sodium hypochlorite. Third, the disinfected bulbs were crushed into a fine powder using a sterilized mortar. One gram sample was mixed with 9 mL sterile water and vortexed to ensure homogeneity. Fourth, the mixture was serially diluted to achieve concentrations of 10^−6^, 10^−7^, 10^−8^, and 10^−9^, and 100 μL aliquots of each dilution was spread onto solid Luria-Bertani (LB) medium. Finally, these LB plates were incubated at 30°C for 48 h. Distinct single colonies were selected for further isolation and purification, culminating in the successful isolation of the *B. velezensis* BN.

The culture conditions for the *B. velezensis* BN were as follows: LB medium with the NaCl concentration adjusted to 0.17 M, the pH maintained at 7.0, and the temperature at 30°C. The cells were observed using a fluorescence microscope (Yongxing N-800F, China).

### 2.2 Antifungal activity of BN strains

To evaluate the antifungal activity of *B. velezensis* BN against diverse fungal pathogens, a dual-culture experiment was conducted, as previously described (Liu et al., 2024). The study encompassed six distinct fungal species: *Colletotrichum gloeosporioides* (*C. gloeosporioides*), *Fusarium oxysporum* (*F. oxysporum*), *Rhizoctonia solani (R. solani*), *Phytophthora infestans* (*P. infestans*), *Fusarium graminearum* (*F. graminearum*) and *Fusarium tricinctum* (*F. tricinctum*). Following a 7 days incubation, the pathogens were inoculated at the center of the Potato Dextrose Agar (PDA) plates. The PDA medium was prepared with 200 g L^−1^ potato, 20 g L^−1^ glucose, and 20 g L^−1^ agar, with the pH adjusted to 7.0. *B. velezensis* BN was inoculated ~1 cm from the edge of each plate, with control plates containing only the pathogens. Each experiment was replicated three times to ensure statistical robustness. All plates were incubated at 28°C until the fungal growth in the control plates extended to the edges.

### 2.3 DNA extraction and 16s rRNA analysis

The genomic DNA of *B. velezensis* BN was extracted using a bacterial DNA extraction kit (General, Shanghai, China). The 16S rRNA gene was amplified using primers 27F (5′-AGAGTTTGATCMTGGCTCAG-3′) and 1492R (5′-GGYTACCTTGTTACGACTT-3′). Sequencing of the PCR products was performed by Sangon Biotech Co., Ltd., (China). The phylogenetic analysis of the 16S rRNA was conducted using the Type (Strain) Genome Server (TYGS) (https://tygs.dsmz.de/) (Le Han et al., 2022; Meier-Kolthoff and Göker, 2019).

### 2.4 Whole genome sequencing and analysis

Whole-genome sequencing of *B. velezensis* BN was performed at Majorbio Laboratory (Shanghai, China) and involved both Scaffolding and completing genome assemblies. For scaffolding, an Illumina paired-end (PE) library with an average insert size of ~400 bp was constructed and sequenced using the Illumina HiSeq platform. This process included *de novo* genome assembly to obtain preliminary genomic sequences and functional annotation. To refine the genome assemblies, a PacBio RS II library with a read length of ~10 kb was generated and sequenced on the PacBio RS II platform, followed by comprehensive bioinformatics analysis. Scaffolding was performed using SOAPdenovo2 software for the assembly of multiple short reads, while completion of genome assemblies were achieved with Unicycler v0.4.8 and Pilon v1.22 for sequence polishing.

Phylogenetic analysis of the *gyrA* gene and whole-genome sequences were conducted using the TYGS server (Le Han et al., 2022; Meier-Kolthoff and Göker, 2019). Average nucleotide identity (ANI) analysis was performed using the JSpeciesWS53 web server (https://jspecies.ribohost.com/jspeciesws/) (Richter et al., 2016). dDDH analysis was conducted using the Genome-to-Genome Distance Calculator 3.0 (GGDC) web server (https://ggdc.dsmz.de/ggdc.php#) (Meier-Kolthoff et al., 2013). Collinearity analysis of the four strains was carried out with Mauve software. Core and pan-genome analyses were performed using BPGA v1.3 software.

### 2.5 Genome annotation and metabolite analysis

Functional annotation of the predicted coding genes was accomplished through a dual-pronged approach. Initially, we aligned the predicted genomic data with the genomes of the *B. velezensis* species, selected as reference genomes based on a phylogenetic analysis of 16S rRNA. This analysis facilitated the identification of homologous genes and their subsequent functional annotation. Subsequently, a database comparison method was employed, leveraging six major databases (NR, Swiss-Prot, Pfam, EggNOG, GO, and KEGG) to enhance the precision of functional descriptions for gene annotation.

Carbohydrate active enzymes were identified by the CAZy database (http://www.cazy.org/). Genes implicated in plant growth-promoting traits were analyzed by PGPT-Pred (https://plabase.cs.uni-tuebingen.de/pb/form.php?var=PGPT-Pred) (Patz et al., 2021). Furthermore, AntiSMASH 7.0 (https://antismash.secondarymetabolites.org/) (Blin et al., 2023) was employed to identify and characterize the biosynthetic gene clusters of secondary metabolites with molecular weights <2.5 kDa.

## 3 Results

### 3.1 Isolation, characterization and preliminary dentification of strain BN

A bacterial strain isolated from the rhizosphere of *Lilium brownii* (lily) was designated as BN. Lily is widely recognized for its various health benefits, such as having calming properties, skin soothing effects, moisturizing dryness, and relieving cough. Additionally, research has shown that Lilium polysaccharides exhibit a variety of pharmacological activities, including antitumor, immunomodulatory, hypoglycemic, and radioprotective properties (Guo et al., 2024). The optimal culture conditions of BN were determined to be a temperature of 30°C, pH 7.0, and 0.17 M NaCl. BN is a Gram-positive bacterium, rod-shaped bacterium with length of 1–3 μm (Supplementary Figure S1A). It is characterized the formation of a substantial biofilm, which may potentially facilitate the colonization in the plant rhizosphere (Paula et al., 2020) (Supplementary Figure S1B).

**Figure 1:**
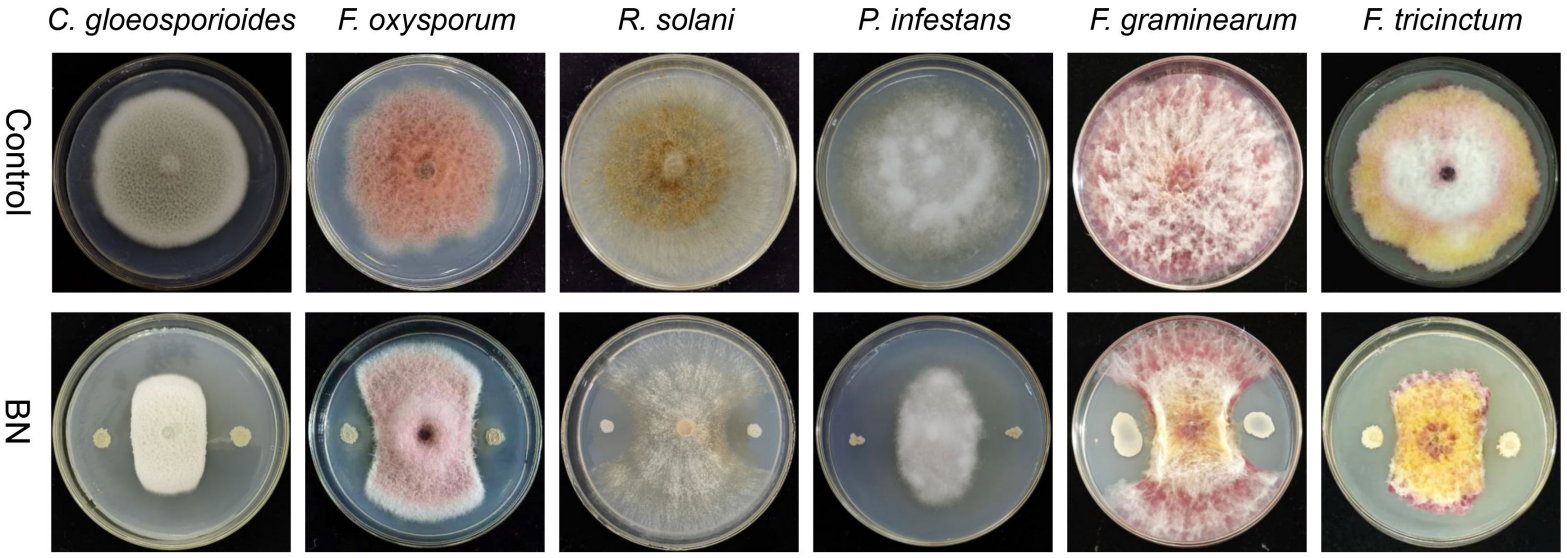
The dual confrontation between BN strain and five fungal pathogens. *C. gloeosporioides, Colletotrichum gloeosporioides*; *F. oxysporum, Fusarium oxysporum*; *R. solani, Rhizoctonia solani*; *P. infestans, Phytophthora infestans*; *F. graminearum, Fusarium graminearum*; *F. tricinctum, Fusarium tricinctum*.

Our study shown that BN exhibits a broad-spectrum antagonistic activity against several fungal plant pathogens, including *C. gloeosporioides, F. oxysporum, R.solani, P. infestans, F. graminearum* and *F. tricinctum* (Figure 1). *C. gloeosporioides* is a globally widespread plant pathogen that causes anthracnose in various plants, including wolfberry, legumes, and strawberries, leading to lesions on fruit and leaf surfaces (Dean et al., 2012). *F. oxysporum* is a destructive soil-borne pathogen that causes fusarium wilt and ceitocybe bescens in over 100 crops, including those in the Musaceae, Rosaceae, and Cucurbitaceae families (Berrocal-Lobo and Molina, 2008; Edel-Hermann and Lecomte, 2019). *R. solani* is a challenging pathogen to control which causes significant damage to a wide range of vegetables, fruits, and crops like potatoes, rice, corn, and cotton, leading to substantial yield and economic losses (Singh A. et al., 2023). *P. infestans* is responsible for causing the serious solanaceous plant disease known as late blight, particularly affecting potatoes, and was a major culprit in the 1840s European (Nowicki et al., 2012). *F. graminearum* is the primary causative agent of Fusarium head blight (FHB), one of the most destructive diseases affecting cereal crops worldwide (Dash et al., 2012; Lu et al., 2022). *F. tricinctum* is a ubiquitous and significant plant pathogen and mycotoxin producer that affects a variety of crops, notably causing Fusarium head blight (FHB) and ceitocybe bescens. In lilies, *F. tricinctum* can result in root rot, bulbs rot, and the yellowing and shedding of basal leaves (Li et al., 2013; Wang Y. et al., 2022). In summary, the BN strain shows promising potential as a highly effective broad-spectrum biocontrol agent.

**Figure 2:**
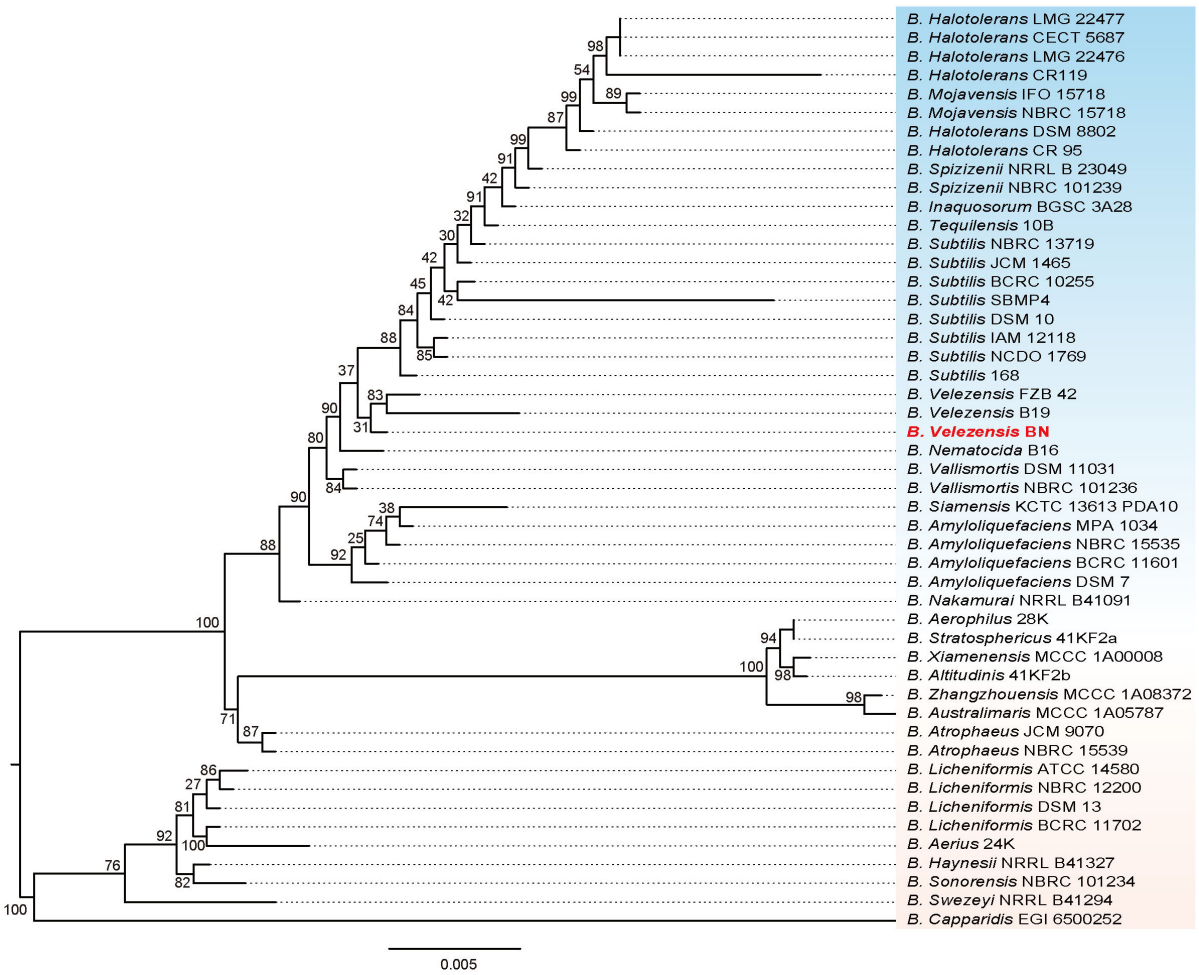
The phylogenetic tree based on 16S rRNA genes illustrates the relationship between the BN strain and other related strains. The scale bar represents 0.005 substitutions per nucleotide position. Additionally, the tree is midpoint-rooted.

We performed a phylogenetic analysis on the 16S rRNA gene sequence of the BN strain (Figure 2). The result revealed that BN exhibited the highest similarity with *B. velezensis* FZB 42, with a 16S rRNA sequence identity of 99.74%. *B. velezensis* FZB 42 serves as the model strain for promoting plant growth and biocontrol rhizosphere (Fan et al., 2018). Additionally, BN shared a sequence identity of 98.84% with *B. velezensis* B19. In summary, based on the sequence similarity of the 16S rRNA genes, coupled with the clustering patterns observed on the phylogenetic tree, BN was preliminarily identified as *B. velezensis*. The complete genome of BN was sequenced to further explore the antimicrobial capabilities and identify additional resistance genes, laying a foundation for developing engineered biocontrol strains.

### 3.2 Genome sequencing of BN and comparative genomics

**Figure 3:**
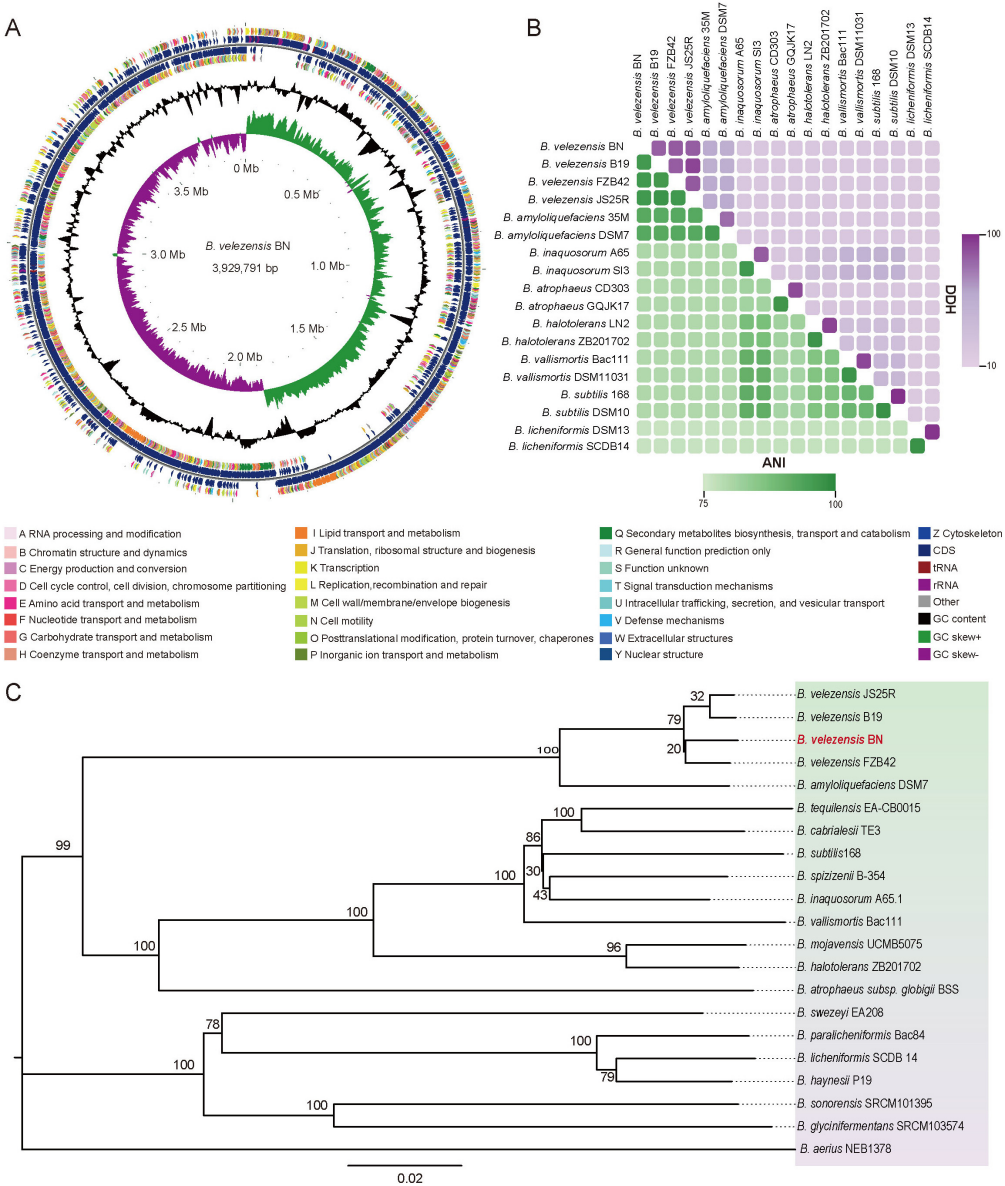
The whole-genome sequencing of BN strain and comparative analysis. **(A)** The circular representation of the BN strain genome. From outer to inner, the first and fourth circles represent the CDS on the positive and negative strands, respectively, with different colors denoting various COG functional. The second and third circles illustrate the CDS, tRNA, and rRNA on the positive and negative strands, respectively. The fifth circle displays the GC content, with outward-facing sections indicating regions where the GC content exceeds the average. The sixth circle illustrates the GC-Skew values. **(B)** The comparisons of ANI and dDDH values of BN strain and other related strains. Green, ANI. Purple, DDH. **(C)** The phylogenetic tree generated from whole-genome sequencing. The scale bar indicates 0.02 substitutions per nucleotide position, and the tree is midpoint-rooted for clarity.

The circular genome map of BN was constructed using the GC view program, as shown in Figure 3A (Grant and Stothard, 2008; Le Han et al., 2022). The whole genome sequence of BN consists of a circular chromosome spanning 3,929,791 bp, with a G+C content of 46.5%, and no plasmid sequences were detected. A total of 3,747 protein-coding genes (CDS) were predicted, accounting for 88.44% of the genome. These CDS genes have an average G+C content of 47.34% and an average length of 928 bp, inclusive of 281 pseudogenes. Additionally, the genome contains 81 small RNAs (sRNAs), 27 rRNAs (nine copies each of 5S, 16S, and 23S rRNAs), and 86 tRNAs.

The ANI and dDDH analyses are widely used to evaluate the similarity across bacterial strains based on whole-genome sequences. According to studies by Zhang et al. (2016), the compared strains exhibiting ANI values of ≥96% and DDH values of ≥70% are typically classified within the same species. In this study, the ANI values between BN and *B. velezensis* FZB 42, *B. velezensis* B19, and *B. velezensis* JS25R were 98.3%, 98.1%, and 98.2%, respectively (Figure 3B). Correspondingly, the DDH values were 85.4%, 83.4%, and 84.5% (Figure 3B).

The phylogeny based on the *gyrA* gene sequence from whole-genome sequences (Supplementary Figure S2), revealing that BN exhibited 98.76% and 98.33% sequence identity with the *gyrA* sequences of *B. velezensis* FZB 42 and *B. velezensis* B19, respectively. The *gyrA* gene, a highly conserved housekeeping gene in the *Bacillus* genus, exists as a single copy across all bacterial species, providing a higher resolution for *Bacillus* identification than the 16S rRNA gene, which may exhibit heterogeneity due to the multicopy (Liu et al., 2022). Furthermore, a phylogenomic tree was constructed based on the complete genome of BN and the published whole-genome sequences of other strains (Figure 3C). Collectively, these data fully confirmed the classification of the BN strain as a member of *B. velezensis* (Figures 2, 3, Supplementary Figure S2).

Comparative genomic analysis using whole genome sequencing with the Mauve program demonstrated that *B. velezensis* BN shares a high degree of homology with the majority of genes from *B. velezensis* FZB 42, *B. velezensis* B19, and *B. velezensis* JS25R. However, compared to *B. velezensis* FZB 42 and *B. velezensis* JS25R, certain genes were found to be absent in *B. velezensis* BN. Additionally, in comparison to *B. velezensis* B19, both deletion and inversion of specific genes were observed in *B. velezensis* BN (Supplementary Figure S3A). Core pan-genomic analysis revealed that the four strains collectively possess 3,266 core genes, constituting 87.2% of the total gene count of *B. velezensis* BN, further substantiating the high similarity between *B. velezensis* BN and the other three strains. Additionally, *B. velezensis* BN contains 111 unique genes (Supplementary Figure S3B).

### 3.3 Metabolite analysis of *B. velezensis* BN

COG (Clusters of Orthologous Groups) gene annotation reveals that the largest proportion of genes fall into three categories: Amino acid transport and metabolism, Transcription, and Carbohydrate transport and metabolism (Supplementary Figure S4A). Notably, genes associated with secondary metabolite biosynthesis, transport, and catabolism constitute 2.1% of the total coding genes.

Carbohydrate active enzymes (CAZy) are a crucial class of enzymes, classified into glycoside hydrolases (GHs), glycosyl transferases (GTs), polysaccharide lyases (PLs), carbohydrate esterases (CEs), Carbohydrate-Binding Modules (CBMs) and Auxiliary Activities (AAs). These enzymes are responsible for degrading, modifying, and generating glycoside bonds. The ability of CAZy to degrade carbohydrates not only provides a competitive advantage against other bacteria but also activates the immunity of host cell (Bu et al., 2023; Wardman et al., 2022). For instance, endophyte-derived α-mannosidase can activate the host immunity by degrading the rice cell wall and releasing oligosaccharides as DAMPs (damage-associated molecular patterns), thereby enhancing the rice's disease resistance (Bu et al., 2023). The function of the *B. velezensis* BN gene was annotated based on the CAZy database (Supplementary Figure S4B). The analysis identified 128 of CAZy, including 41 GHs, 42 GTs, two CBMs, 31 CEs, three PLs, and nine AAs.

**Figure 4:**
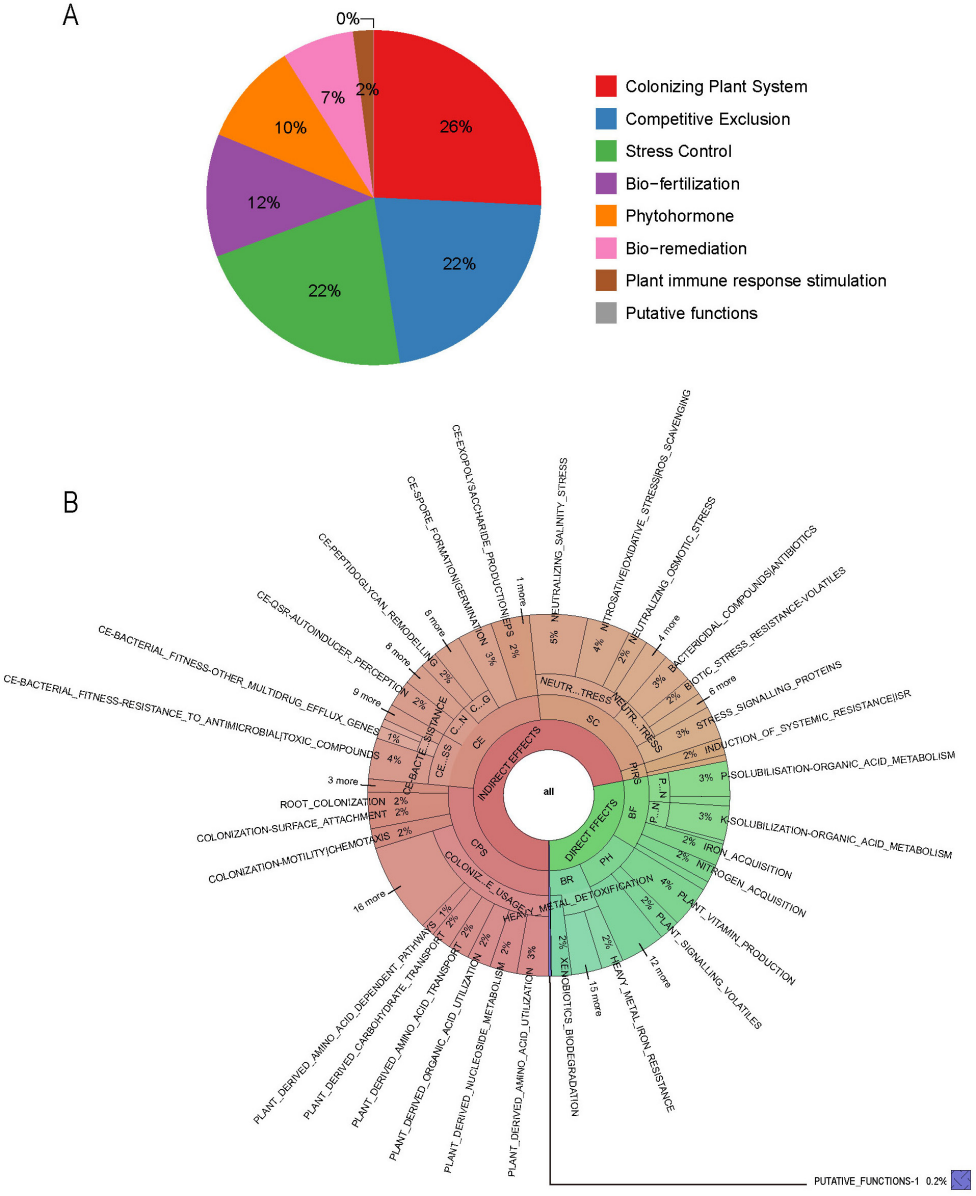
The functional genes of *B. velezensis* BN associated with plant growth-promoting traits. **(A)** The proportion of various functional genes in the genome. **(B)** Function genes that directly or indirectly affect plant growth. CPS, colonizing plant system; CE, competitive exclusion; SC, stress control; PIRS, plant immune response stimulation; BF, bio-fertilization; PH, phytohormone; BR, bio-remediation.

PGPT-Pred is an analytical tool designed to predict plant growth-promoting traits within single bacterial genomes (Patz et al., 2021). As of the analysis conducted in August 2024, PGPT-Pred indicates that the gene distribution within *B. velezensis* BN is as follows: 26% are associated with plant colonization systems, 22% with competitive exclusion, 22% with stress control mechanisms, 12% with bio-fertilization, 10% with phytohormones, 7% with bioremediation, and 2% with plant immune response stimulation (Figure 4A). Notably, genes involved in plant colonization, competitive exclusion, and stress control, as well as plant immune response stimulation, indirectly influence plant growth, while those associated with bio-fertilization, phytohormone, and bio-remediation have a direct effect on promoting plant growth (Figure 4B, Supplementary Figure S5).

### 3.4 Secondary metabolites analysis of *B. velezensis* BN

Biocontrol strains can produce a variety of secondary metabolites with diverse properties and structures, exhibiting a broad range of activities. These secondary metabolites include antibiotics, pigments, plant growth promoters, effectors of ecological competition, enzyme inhibitors, and pheromones (Chaabouni et al., 2012). Among them, compounds with a molecular weight below 2.5 kDa, produced during the stationary phase, represent the most important class of secondary metabolites for the control of plant diseases (Jayakumar et al., 2011; Ruparelia et al., 2022). They exhibit a wide range of antibacterial properties, triggering systemic acquired resistance, and promoting biofilm formation which facilitates the colonization for the producing strains. The antiSMASH platform is the leading resource for identifying and analyzing the biosynthetic gene clusters (BGCs) responsible for compounds with a molecular weight below 2.5 kDa in both archaea and bacteria (Blin et al., 2023).

The antiSMASH was employed to predict the BGCs in *B. velezensis* BN, aiming to assessing its capacity to suppress pathogenic activity. The analysis revealed a total of 12 BGCs dedicated to the production of compounds with a molecular weight below 2.5 kDa (Table 1), spanning a combined length of 754.8 kb, which constitutes a significant 19.2% of the genome's total length. Among the identified BGCs are two encoding nonribosomal peptide synthetases (NRPS), two trans-Acyl transferase polyketide synthases (transAT-PKS), one type III polyketide synthase (T3PKS), two transAT-PKS-NRPS hybrid systems, one lanthipeptides, one putative polyketide synthase (PKS), two terpene clusters, and one cluster of an undetermined type (Table 1).

**Table 1:** Prediction of biosynthetic gene clusters of secondary metabolites in *B. velezensis* BN using antiSMASH 7.0.

**Region**	**Type**	**Start**	**End**	**Most similar known cluster**	**Similarity**	**Formula**	**MIBiG accession**	**Gene cluster from organisms**
Region 1	NRPS	322,199	387,606	Surfactin	82	C_52_H_91_N_7_O_13_	BGC0000433	*Bacillus velezensis* FZB42
Region 2	PKS-like	924,056	965,300	Butirosin A/ButirosinB	7	C_21_H_41_N_15_O_12_	BGC0000693	*Bacillus circulans*
Region 3	Terpene	1,047,344	1,068,084					
Region 4	Lanthipeptide-class-II	1,188,577	1,217,465					
Region 5	TransAT-PKS	1,384,028	1,472,261	Macrolactin H	100	C_24_H_34_O_5_	BGC0000181	*Bacillus velezensis* FZB42
Region 6	TransAT-PKS, T3PKS, NRPS	1,690,940	1,801,045	Bacillaene	100	C_34_H_50_N_2_O_6_	BGC0001089	*Bacillus velezensis* FZB42
Region 7	NRPS, TransAT-PKS, betalactone	1,865,676	2,003,477	Fengycin	100	C_72_H_110_N_12_O_20_	BGC0001095	*Bacillus velezensis* FZB42
Region 8	Terpene	2,028,704	2,050,587					
Region 9	T3PKS	2,113,905	2,155,005					
Region 10	TransAT-PKS	2,269,991	2,376,173	Difficidin	100	C_31_H_45_O_6_P_1_	BGC0000176	*Bacillus velezensis* FZB42
Region 11	NRP-metallophore, NRPS, RiPP-like	3,000,877	3,052,668	Bactillihactin	100	C_39_H_42_N_6_O_18_	BGC0000309	*Bacillus subtilis* 168
Region 12	Other	3,588,978	3,630,396	Bacilysin	100	C_12_H_18_N_2_O_5_	BGC0001184	*Bacillus velezensis* FZB42

**Figure 5:**
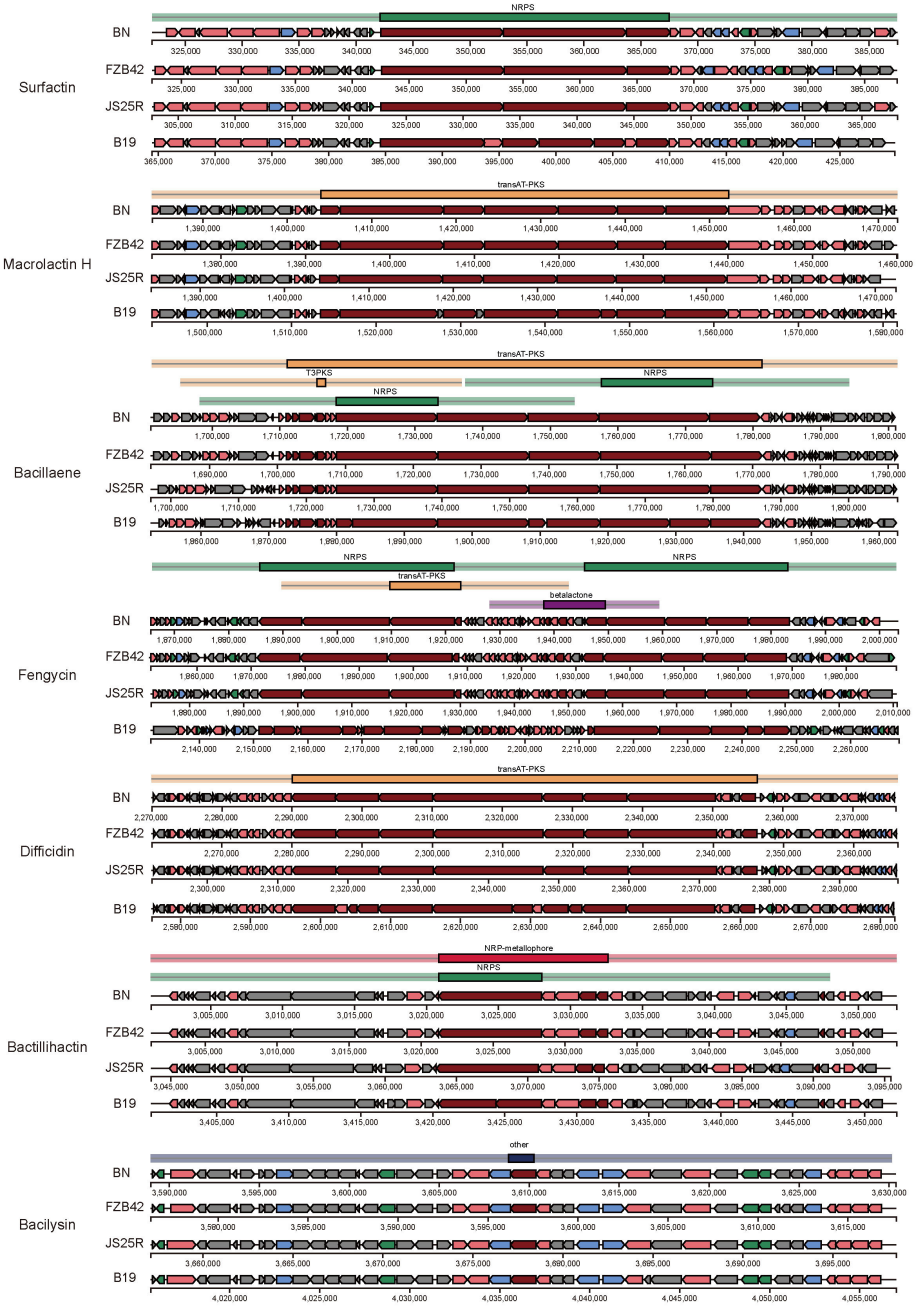
Comparison of the secondary metabolite biosynthetic gene clusters among *B. velezensis* BN and three other *B. velezensis* strains. BN, *B. velezensis* BN. FZB42, *B. velezensis* FZB42. JS25R, *B. velezensis* JS25R. B19, *B. velezensis* B19.

Further analysis revealed that seven BGCs in *B. velezensis* BN exhibit a high degree of similarity to those found in the model strains *B. velezensis* FZB 42 and *B. subtilis* 168. The sequences of macrolactin H, bacillaene, fengycin, difficidin, bactillihactin, and bacilysin are 100% homologous with the reference strains, while surfactin show 82% similarity based on alignment with the public database (Table 1). Additionally, we compared the sequence differences of seven compounds among four strains: *B. velezensis* BN, *B. velezensis* FZB 42, *B. velezensis* JS25R, and *B. velezensis* B19. The results demonstrate that the four strains possess similar core biosynthetic genes, but *B. velezensis* B19 exhibits some differences compared to the other three (Figure 5), which aligns with the findings of the collinearity analysis (Supplementary Figure S3A). These differences may be attributed to the distinct environmental in which these strains evolved, leading to the development of unique metabolic pathways as adaptive mechanisms.

In this study, we conducted an antiSMASH analysis to provide a detailed summary of several key compound biosynthetic pathways (Figure 5). The biosynthetic gene cluster for surfactins is composed of four open reading frames (ORFs): *licA, licB, licC*, and *srfATE*, with a total length of 26.1 kb. In other species, the gene cluster ORFs related to surfactin biosynthesis are named *srfAA, srfAB, srfAC*, and *srfAD* (Ongena and Jacques, 2008). The synthesis of macrolactin H is catalyzed by a PKS encoded by seven genes, *pksE, mlnB, mlnC, mlnD, mlnE, mlnF*, and *mlnG*, totaling 48.2 kb. The biosynthesis of bacillaene involves a PKS composed of *pksC, pksD, pksE, acpK, pksG, pksH, pksI*, and *pksL*, along with a NRPS composed of *pksJ, pksN*, and *pksR*, resulting in a total gene cluster length of 70.1 kb. Fengycin is catalyzed by a NRPS composed of *ppsA*-*E*, with a total gene cluster length of 37.7 kb. Difficidin is catalyzed by a PKS composed of *dfnD*-*J*, totaling 60 kb. Bactillihactin comprises five ORFs, *entA, entC, entE, entB*, and *dhbF*, with a total length of 11.7 kb. In addition, bacillibactin is a siderophore that can absorb iron elements at extremely low iron concentrations. Given that iron atoms in nature primarily exist as ferric iron, which has low bioavailability at physiological pH, the role of bacillibactin is particularly crucial (Jia et al., 2021; Ma et al., 2017). The bacilysin is primarily catalyzed by a series of enzymes encoded by *bacA*-*G*, each performing distinct functions. The biosynthetic pathways of these natural active substances not only reveal their roles in microbial metabolism but also provide a potential for constructing artificial biocontrol engineering strains.

## 4 Discussion

The PGPR confer benefits to plants by inhibiting pathogenic invasion and facilitating the acquisition of soil nutrients, thereby serving as an effective alternative to chemical agents in the prevention and control of diseases and pests (Ling et al., 2022). *Bacillus* species are characterized by their exuberant metabolism, production of diverse secondary metabolites, high fermentation density, and strong resistance and adaptability, which render an important group of beneficial biocontrol strains in nature environment (Fira et al., 2018). In recent years, there has been a notable increase in the discovery of *B. velezensis*. As of August 2024, the genomes from 928 strains of *B. velezensis*, either fully or partially, have been submitted to the National Center for Biotechnology Information (NCBI) database. This repository is pivotal for the exploration of antimicrobial compounds and the development of biocontrol engineering strains that can be artificially controlled.

In this study, we isolated a novel PGPR from the rhizosphere of lily, which thrives at growth temperature of 30°C, pH 7.0, and 0.17 M NaCl. Phylogenetic analysis based on 16S rRNA, *gyrA* gene and whole genome sequence revealed that this strain is a member of the *B. velezensis* species (Figures 2, 3C, Supplementary Figure S2). We found that it exhibits significant antimicrobial activity against various phytopathogenic fungi, including *C. gloeosporioides, F. oxysporum, R. solani, P. infestans, F. graminearum*, and *F. tricinctum* (Figure 1). Therefore, *B. velezensis* BN emerges as a promising candidate for biopesticide applications, offering a potential solution for the prevention and management of plant diseases caused by fungal pathogens.

In PGPR, secondary metabolites with molecular weights below 2.5 kDa are identified as the most critical antagonistic substance. Among these compounds, fengycins and surfactins, which are cyclic lipopeptides, distinguished by their unique composition of both amino acid and hydroxy fatty acid chains (Supplementary Figures S6A, B). Their exhibit significant heterogeneity, which can be attributed to the diversity in the type and sequence of amino acid residues, the unique feature of peptide fragment cyclization, and the unique characteristics of hydroxy fatty acid chains regarding their length, branching, and the specific synthetic pathways involved. The broad-spectrum antibacterial properties of fengycins and surfactins have positioned them as potent agents in the management of plant disease (Ongena and Jacques, 2008). In recent study, we found that elevating surfactins levels in *B. velezensis* BN not only thickens biofilm formation but also enhances its colonization capacity on plant roots (unpublished data).

Macrolactin, a member of 24-membered macrolides (Supplementary Figure S6C), is renowned for the robust antibacterial potency. Studies have demonstrated its efficacy in neutralizing plant pathogens such as *Alternaria spp*. and *Pyricularia oryzae*, highlighting its significant potential in agricultural disease and pest management (Wu et al., 2021; Xue et al., 2008). Bacillibactin, a siderophore (Supplementary Figure S6D), plays a pivotal role in iron acquisition, especially under iron-deficient conditions. Notably, Chakraborty et al. (2022) has indicated bacillibactin as a promising antimicrobial agent. Difficidin, a highly unsaturated 22-membered macrolide phosphate, and bacilysin, a dipeptide antibiotic with an L-alanine residue at the N-terminal and the non-protein amino acid L-anticapsin at the C-terminal (Andrić et al., 2023; Özcengiz and Ögülür, 2015) (Supplementary Figures S6E, F), have been established in previous research to possess potent antibiotic properties, particularly against the human pathogen *Candida albicans* (Özcengiz and Ögülür, 2015). Recent studies have further revealed that they can modulate the expression of genes associated with virulence, cell division, and the synthesis of proteins and cell walls in *Xanthomonas*, pathogens responsible for rice white leaf blight and bacterial stripe disease (Chen et al., 2019). Bacillaenes, highly conjugated linear polyene with several cis-double bonds (Supplementary Figure S6G), exhibit broad antibacterial activity and are crucial for the strains survival, competition with other microorganisms, and biofilm formation (Miao et al., 2023). Bacillaene has been shown to assist *B. velezensis* FZB 42 in effectively inhibiting *E. amylovora*, the pathogen causing severe fire blight in apples and pears (Chen et al., 2009).

Although PGPR hold substantial potential as alternatives to chemical pesticides, their successful commercial applications face challenges due to species exclusion and the complexities of adapting to diverse ecological niches (Shade et al., 2012). Consequently, developing eco-friendly biopesticides requires both identifying novel, broad-spectrum antimicrobial strains and genetically modifying these strains to enhance their adaptability to fluctuating ecological conditions. Synthetic biology, a burgeoning field, employs engineering principles to redesign biological systems, thereby enabling the endowment functionalities in organisms through gene editing, gene synthesis, genetic circuits construction, and metabolic engineering (Khalil and Collins, 2010). The field is increasingly being harnessed in the development of novel biopesticides, as evidenced by the microbial synthesis of plant-derived compounds such as emodin and celangulin (Ke et al., 2021; Zhao et al., 2022). Ginkgo Bioworks has capitalized on synthetic biology to engineer efficient plant protectants by optimizing microbial production of potent insecticidal compounds for large-scale application.

Furthermore, the NRPS engineering has been a prominent area of research in recent years. By rearranging NRPS modules with various combinations, it is anticipated that new non-ribosomal peptides can be synthesized, thereby expanding the repertoire of bioactive molecules found in nature. A range of strategies for NRPS reconstruction has been developed, including the specific adjustments of adenylation domain (Stachelhaus et al., 1995), subdomain swaps, docking domain engineering (Mootz et al., 2002), and the insertion or deletion of modules/domains (Butz et al., 2008; Mootz et al., 2002). By deleting and replacing docking domains, mutating key amino acids, and using the same docking domain for multiple NRPS subdomain, the biosynthetic pathways for surfactin and fengycin have been successfully engineered, leading to the detection of products with altered chain lengths in the host cells (Liu et al., 2016; Mootz et al., 2002). The XUT method facilitates the efficient recombination of thioesterase domains from different species, enabling the targeted design and synthesis of peptides with specific bioactivities (Bozhüyük et al., 2024). Präve et al. (2024) successfully developed a novel syrbactin derivative that selectively inhibits the activity of the immunoproteasome by the XUT method.

In this study, we have successfully isolated and sequenced the *B. velezensis* BN strain, which provides potential for genetic manipulation to increase the yield of compounds antagonistic to plant pathogens. Additionally, we introduce the feasibility of utilizing synthetic biology techniques to construct and reengineer efficient antimicrobial agents within the chassis cells, such as non-ribosomal peptides. This study lays a foundation for the development of novel microbial pesticides, which are crucial for the sustainable enhancement of plant growth, nutrition, health, disease control, and overall productivity in fluctuating and challenging environmental contexts.

## 5 Conclusion

This study has achieved the isolation a PGPR strain from the lily rhizosphere, which was identified as *B. velezensis*. The complete genome of *B. velezensis* BN consists of a circular chromosome with a length of 3,929,791 bp and a G+C content of 46.5%. Our findings revealed that *B. velezensis* BN exhibits strong antagonism against a range of fungal plant pathogens, thereby positioning it as a PGPR with broad-spectrum antimicrobial activity. Genomic analysis showed that the genome of *B. velezensis* BN is abundant in CAZy genes, conferring a significant competitive advantage in diverse ecological niche. Moreover, genes directly involved in promoting plant growth accounted for 29% of the total gene count, providing a robust basis for plant growth enhancement. Additionally, the genome of *B. velezensis* BN encompasses genes encoding secondary metabolites with molecular weight <2.5 kDa, such as fengycin and surfactin, which account for 19.2% of the total genome and may play a pivotal role in the broad-spectrum antimicrobial activity. This study not only expands our understanding of the PGPR strain *B. velezensis* BN but also offers a scientific foundation and potential application for the development of efficient functional microbial agents and the engineering of artificially controllable biopesticide cell factories.

## Data Availability

The original contributions presented in the study are publicly available. This data can be found here: PRJNA1184975.
