# Supplementary material for: Whole-genome sequencing and secondary metabolite exploration of the novel Bacillus velezensis BN with broad-spectrum antagonistic activity against fungal plant pathogens
Source: Front Microbiol. 2025 Jan 3;15:1498653. doi: 10.3389/fmicb.2024.1498653 (PMC11738913; doi:10.3389/fmicb.2024.1498653)
Supplement: Supplementary file 1 [file Table_1.docx]

**Supplementary materials for**

Whole-genome Sequencing and Secondary Metabolite Exploration of the Novel *Bacillus velezensis* BN with Broad-spectrum Antagonistic Activity Against Fungal Plant Pathogens

Yanli Zheng, Tongshu Liu, Ziyu Wang, Xu Wang, Haiyan Wang, Ying Li, Wangshan Zheng, Shiyu Wei, Yan Leng, Jiajia Li, Yan Yang, Yang Liu, Zhaoyu Li, Qiang Wang, Yongqiang Tian *

* Correspondence: Yongqiang Tian, School of Biological and Pharmaceutical Engineering, Lanzhou Jiaotong University, Lanzhou 730070, Gansu, China, E-mail: tianyq@mail.lzjtu.cn

**This file includes:**

Figure S1 to S6


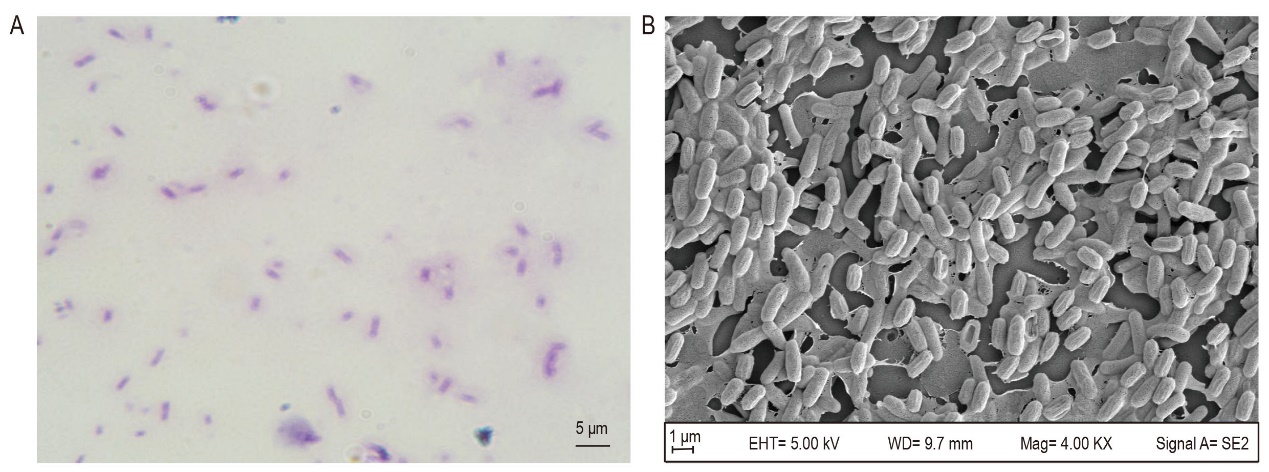


Fig. S1. The morphological characteristics of *B. velezensis* BN, A, Gram staining. B, Observation by scanning electron microscope (ZEISS Ultra Plus, Germany), EHT, accelerating voltage. WD, working distance. Mag, magnification. Signal A=SE2, the E-T detector collects SE2 electrons.


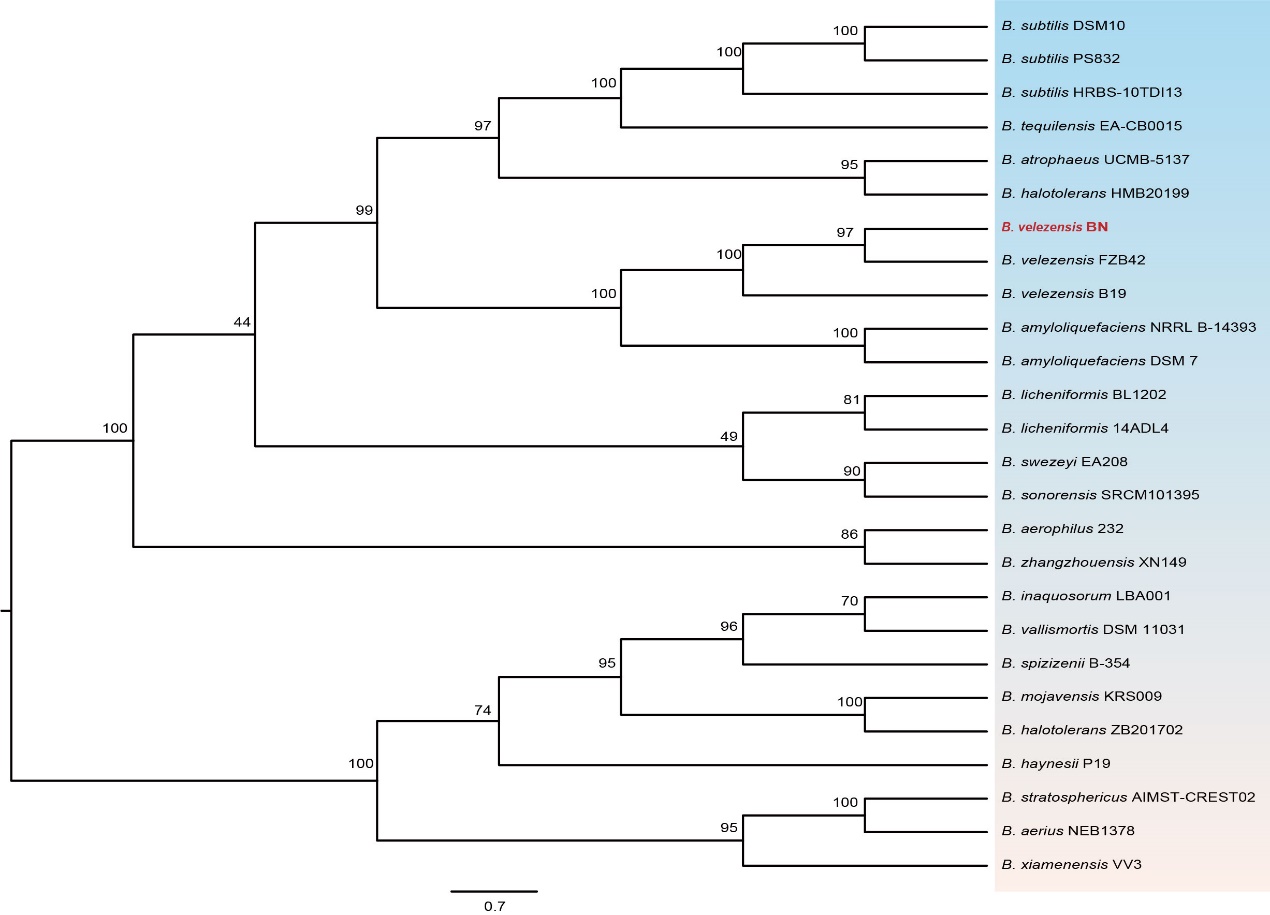


Fig.S2. The phylogenetic tree based on *gyrA* gene sequence. The scale bar represents 0.7 substitutions per nucleotide position. Additionally, the tree is midpoint-rooted.


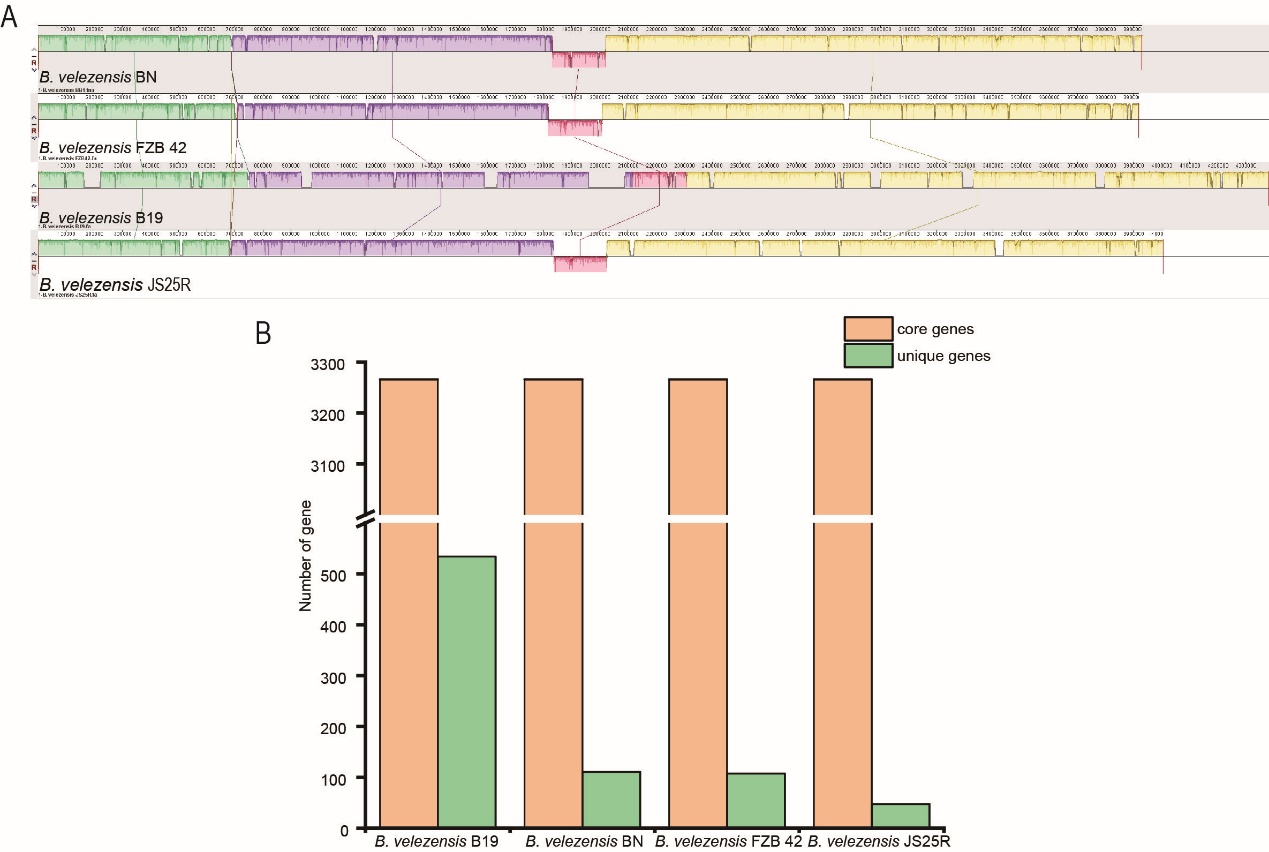


Fig.S3. The comparative genomic analysis of among *B. velezensis* BN and three other *B. velezensis* strains. A, Synteny analysis of *B. velezensis* BN and *B. velezensis* FZB42, *B. velezensis* B19, *B. velezensis* JS25R. B, The number of core genes and unique genes of four strains.


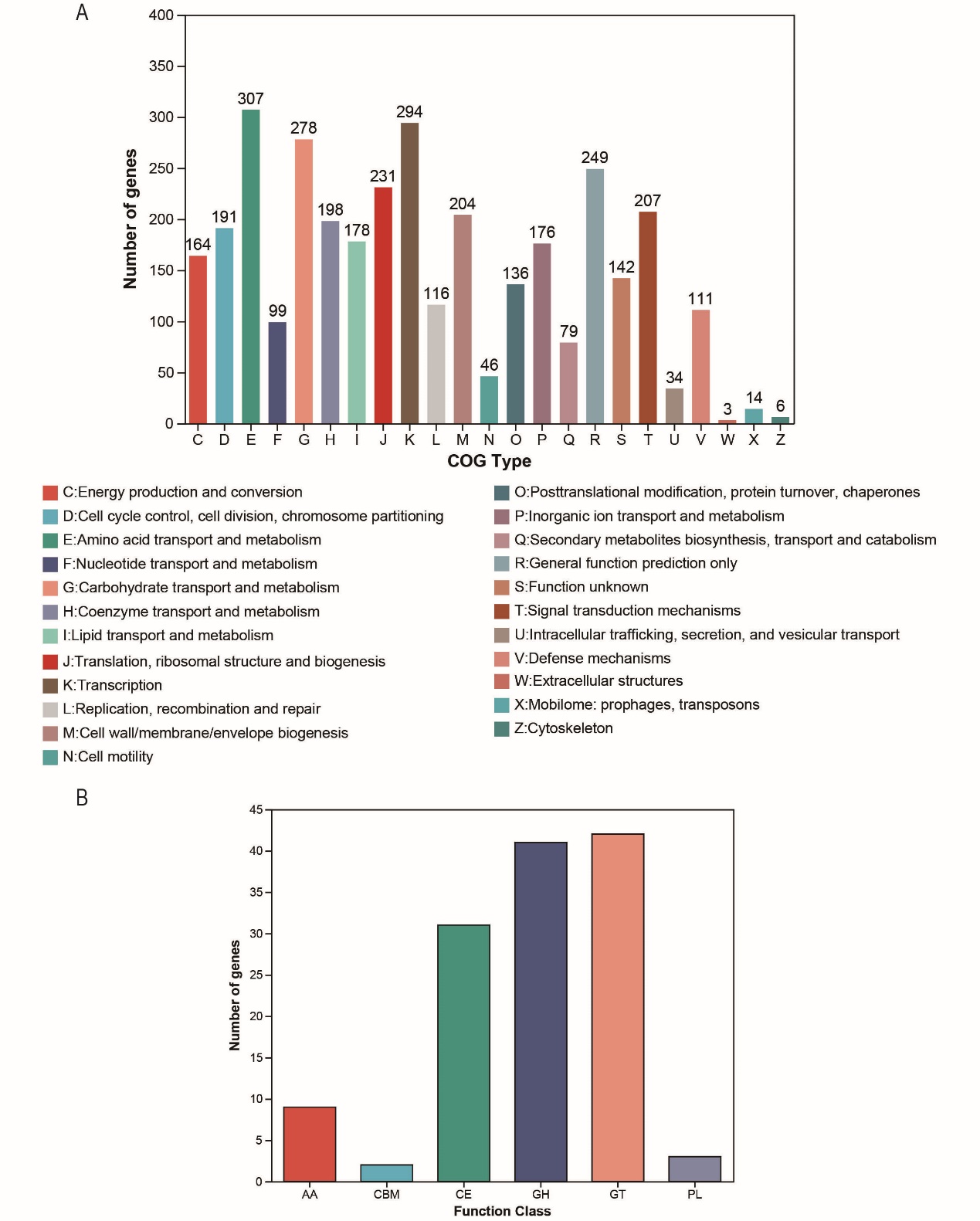


Fig.S4. The metabolite analysis of *B. velezensis* BN. A, COG gene annotation. B, CAZy analysis. AA, Auxiliary Activitie. CBM, Carbohydrate-Binding Module. CE, carbohydrate esterase. GH, glycoside hydrolase. GT, glycosyl transferase. PL, polysaccharide lyase.


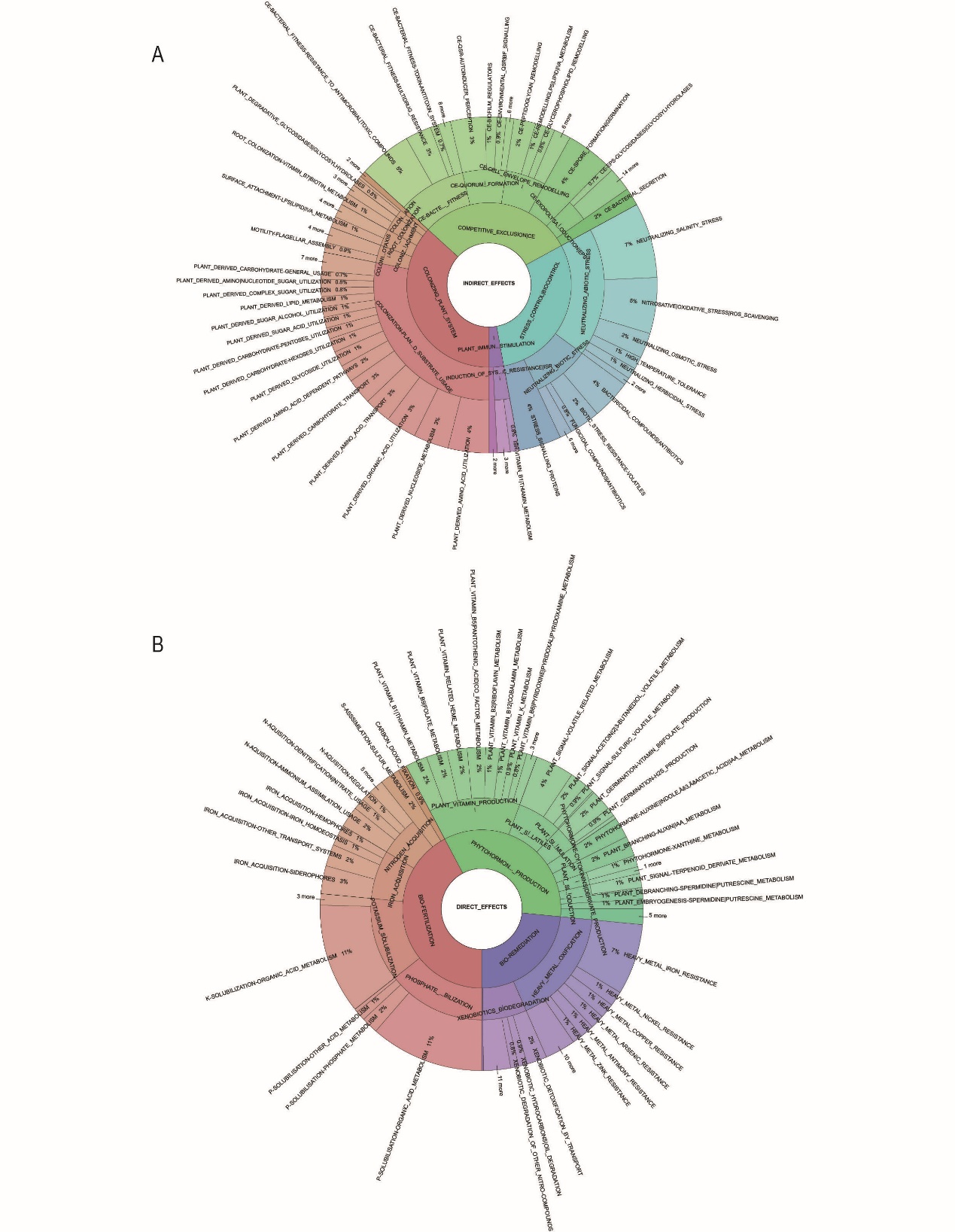


Fig.S5. The functional genes of *B. velezensis* BN that influence plant growth, both indirectly (A) and directly (B).


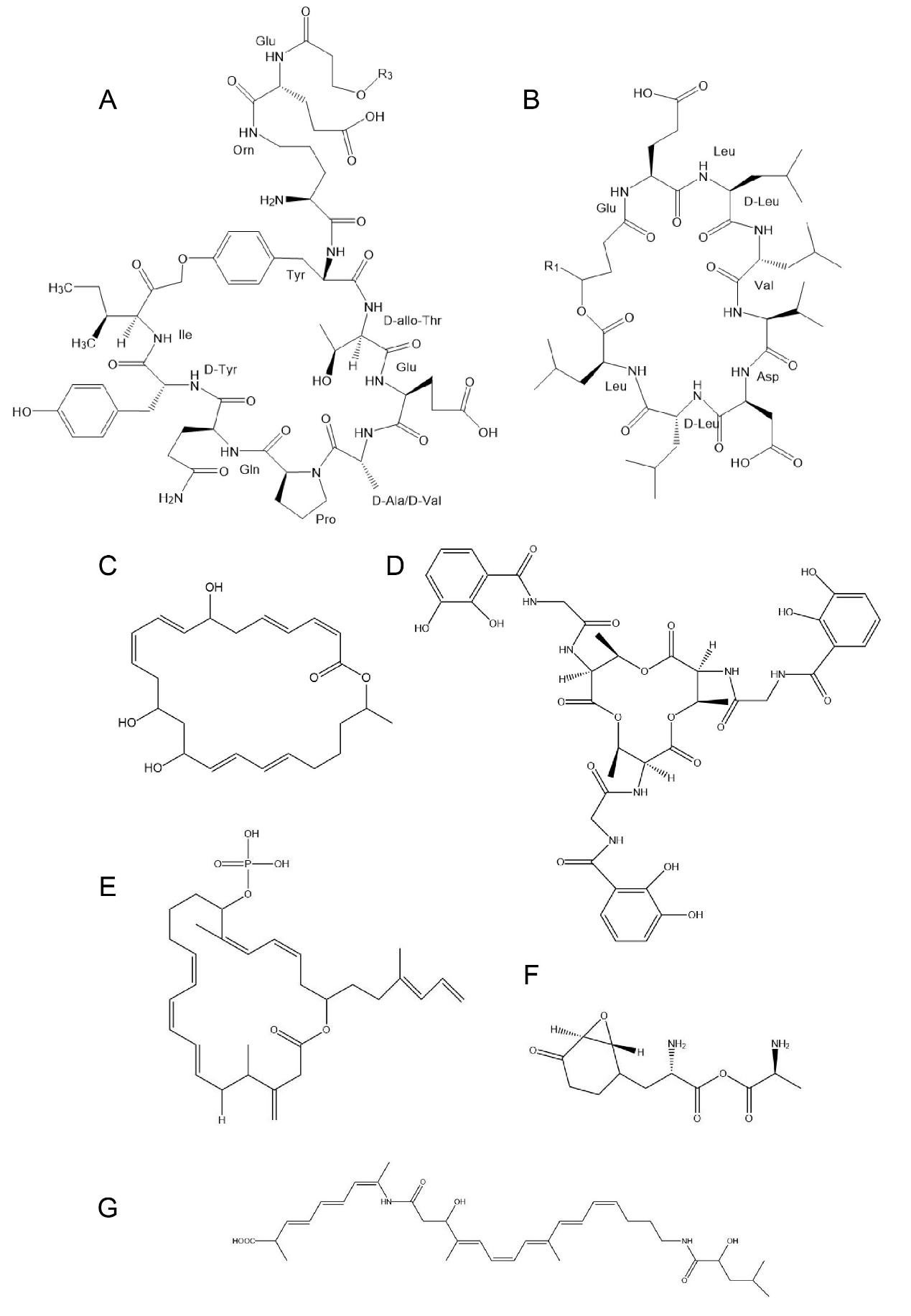


Fig.S6. The structural characteristics of various secondary metabolites. A, Fengycins. B, Surfactins. C, Macrolactin. D, Bacillibactin. E, Difficidin. F, Bacilysin. G, Bacillaenes.
